# Patient-Pathway Analysis of Tuberculosis Services in Cameroon

**DOI:** 10.3390/tropicalmed6040171

**Published:** 2021-09-22

**Authors:** Collins N. Titahong, Gideon N. Ayongwa, Yvonne Waindim, Dubliss Nguafack, Albert Kuate Kuate, Irene Adeline Goupeyou Wandji, Alison Wringe, Vincent Mbassa, Melissa S. Sander, Ellen M. H. Mitchell

**Affiliations:** 1 Tuberculosis Reference Laboratory Bamenda, Center for Health Promotion and Research, Bamenda, Northwest, Cameroon; titahongnosohcollins@gmail.com (C.N.T.); ayongwag@gmail.com (G.N.A.); waindimy@gmail.com (Y.W.); 2 National TB Program, Yaoundé, Center, Cameroon; waksun@gmail.com (D.N.); akuate2001@yahoo.com (A.K.K.); vincentmbassa@yahoo.fr (V.M.); 3 National TB Program-Littoral Region, Douala, Littoral, Cameroon; adywandji@gmail.com; 4 The Global Fund to Fight AIDS Tuberculosis and Malaria, 1218 Geneva, Switzerland; Alison.Wringe@theglobalfund.org; 5 Public Health Department, ITM Antwerp, 2000 Antwerp, Belgium

**Keywords:** tuberculosis, patient-pathway analysis (PPA), care-seeking, Cameroon

## Abstract

In Cameroon, in 2019, tuberculosis (TB) treatment coverage was estimated at 53%, indicating that almost half of all people sick with TB were not diagnosed or linked to care. To inform strategies to improve access to TB services, we conducted an evaluation of the alignment between patient-initiated care-seeking behavior and spatial and institutional allocation of TB services. Data sources included the Cameroon Demographic and Health Survey (2018), the Health Facility List (2017), and routinely collected TB surveillance data. Data visualization was performed in Tableau and QGIS. The pathway analysis showed that only an estimated 9% of people attended a health facility providing TB services at initial care-seeking, with access varying from <3% to 16% across the ten regions of the country. While 72% of government and 56% of private hospitals (Level 2 facilities) provide TB services, most Cameroonians (87%) initially chose primary care (Level 1) or informal private sector sites (Level 0) without TB services. The gaps were greatest in regions with the highest prevalence of poverty, a significant determinant for TB. These results indicate that access may be improved by expanding TB services at both public and private facilities across the country, prioritizing regions with the greatest gaps.

## 1. Introduction

Globally, in 2019, an estimated 2.9 million of the total 10 million people with tuberculosis (TB) were not diagnosed, notified, or linked to TB treatment [1]. Finding and treating people with TB is challenging due to many factors, including non-specific disease presentation, inadequate performance of the currently available rapid diagnostic techniques, and the limited resources of many of the people and countries that are most affected by TB [2]. Access to TB diagnosis and treatment is a major barrier in many settings, and insufficient availability of diagnostic and treatment resources contributes to ongoing transmission and mortality [3]. The demand for TB services is a key parameter for program design, but it is poorly understood, infrequently measured, and commonly under-valued [4,5,6].

Several complementary gap analysis tools have been described to assess the challenges people face in accessing care for TB, including the Onion Model [7], patient care cascades [8], the MATCH approach [9], and Finding all the Missing Persons [10]. Recently, another approach, the TB patient-pathway analysis (PPA), has been recommended by the Lancet Commission [11] and conducted in several countries [12,13,14,15]. The overall goal of the PPA is to identify the gaps between where people initially seek care for TB and where TB services are currently available. The method for producing a PPA has been standardized and typically relies on the use of data that is already available to help countries identify TB service provision gaps [16].

Cameroon has a population of approximately 26 million people and an estimated TB incidence of 179 (range, 116–255) TB cases per 100,000 people was reported in 2019 [1]. The National TB Program supervises diagnostic TB testing and provides free TB treatment at 261 health facilities throughout the country. Although TB case notifications in Cameroon increased from the 1990s until 2014 [17,18], they have been declining since then, in line with the decreasing estimated TB incidence, even though TB treatment coverage remains relatively low, at 53% (range, 35–77%) in 2019 [1].

A better understanding of the alignment between initial patient-initiated care-seeking for TB and the availability of TB diagnostic and treatment centers in Cameroon is critical to improve the spatial allocation of diagnostic and treatment services and to lower patient-borne costs for those at the highest risk of TB. To this end, we conducted a patient-pathway analysis (PPA) following a previously described approach, using available population-based surveys and health systems data [12,16].

## 2. Materials and Methods

We used the approach to construct the patient-pathway analysis for TB services that has been described by Hanson et al. [12] with the Patient-Pathway Analysis: How-to Guide available at http://linksbridge.com/work/tb-ppa/ [16]. Briefly, this approach begins with collecting the most relevant available information at the national and sub-national level on patient care-seeking behavior, health facilities and types, and TB service provision. The data are then organized and standardized across sources by health facility sector and type. This facilitates assessment along the care pathway from initial care-seeking to diagnostic access, treatment access, treatment notification, and treatment outcome. Finally, the data are visualized to enable easier presentation in a standard format of seven columns for the placement of initial care seeking (column 1), coverage of and access to TB diagnostic testing (columns 2/3), coverage of and access to TB treatment (columns 4/5), and location and treatment outcomes of people notified with TB (columns 6/7). Patient-pathway analyses were conducted at the national level and for each of the ten geographical regions in the country.

Three primary data sources were used for the analysis (Table 1). Care-seeking information was obtained from the Cameroon Demographic and Health Survey (DHS 2018) [19]. The DHS 2018 included only limited data for the Southwest region, due to security-related challenges for data collection; for this region, we used data from the DHS 2011 instead. Since questions about the location of care-seeking for adults with signs and symptoms of tuberculosis were not included in the DHS, the place of care-seeking for a child under 5 years old with fever in the two weeks prior to the DHS survey was used as a proxy for TB care-seeking. This approach has been recommended when TB-specific data is unavailable, based on data from countries where the proxy of care-seeking for a child with fever has been found to be comparable to TB care-seeking behavior [13,14,15].

Information about numbers and types of health facilities in the country were obtained from the 2017 Cameroon Health Facility List. Data on the type and level of facilities where TB services were available, where and how many people were notified with TB, and their treatment outcomes were obtained from the National TB Program, with data from 2018 used for the analysis. To enable comparison across the various data sources, we categorized and standardized the health facility type and level for the different data sources (Table S1). The DHS included four sectors of health facility: public sector, private sector, other, and non-governmental organization. For the PPA, the categories of “other” (including informal medicine vendors and traditional practitioners) and “NGO” (including NGO, store, community relay/worker, and other) were grouped together under “informal private”. The Cameroon Health Facility List included 5309 health facilities, with 2484 (47%) public structures and 2825 private structures. The Health Facility List is not comprehensive; in comparison, the Health Map 2016 included 5853 facilities (540 more than the HFL), with 2675 (46%) public and 3178 private facilities [20]. The private facilities in the list include faith-based non-profit, private non-profit, and private for-profit facilities and were combined under the grouping of private facilities for this analysis. For this patient-pathway analysis, the Health Facility List was used as it contained approximately 90% of the health facilities in the Health Map 2016 and also included individual health facility classification that enabled categorization by level as 0, 1, 2, or 3 as recommended in the PPA [16]. In this categorization, Level 0 is community-based, with basic care typically provided by community health workers or extension workers, who are often linked to higher-level health facilities. Level 0 also includes pharmacies and other informal private sector providers. Level 1 facilities provide primary care, typically by a nurse and on an outpatient basis, though higher-level services are available at some sites. Level 2 facilities provide both primary and higher-level care; these include district hospitals and private hospitals (confessional, non-profit, and for-profit). Level 3 facilities provide specialized services and have a larger inpatient capacity, including general and central hospitals. For the patient-pathway analysis, the Level 3 structures in the health facility list have been merged with the Level 2 structures to facilitate alignment with the DHS care-seeking data. The health facilities on the National TB Program service provider list were matched to those in the Cameroon Health Facility List by region and name. The program-supervised facilities were a sub-set of the Health Facility list, with the exception of prisons; the National TB Program supervised four prisons that have both TB diagnostic and treatment services and were not included on the Health Facility List.

For the analysis, the data from each of the various sources were entered as raw and/or summary data into the standardized PPA Excel template, both nationally and for each administrative region, following the steps described in the patient-pathway analysis guide [16]. TB diagnostic and treatment access at the first point of care were calculated by multiplying the percentage of people who reported presenting at each health facility type and level (e.g., 8% of people surveyed reported attending level 2 public facilities) by the percentage of those facilities having TB testing and treatment services (for example, 72% of public level 2 facilities had TB services). In Cameroon, in 2018, all facilities providing TB testing also provided TB treatment, so the coverage of diagnostic services and treatment have been combined in columns 2/4 and 3/5; the combined numbering enables direct comparison with other analyses using this standardized procedure for PPA that have used seven columns. Overall estimates for each level and sector were obtained summing the percentages for each category. The estimates of access to TB services at initial care-seeking were also compared to key information for TB elimination efforts, including the regional wealth distribution, from the DHS 2018 wealth quintile data [19], and to the regional health facility density by population. The summary data were visualized using Microsoft Excel, Tableau, Adobe Illustrator, and QGIS Geographic Information System.

## 3. Results

**Figure 1:**
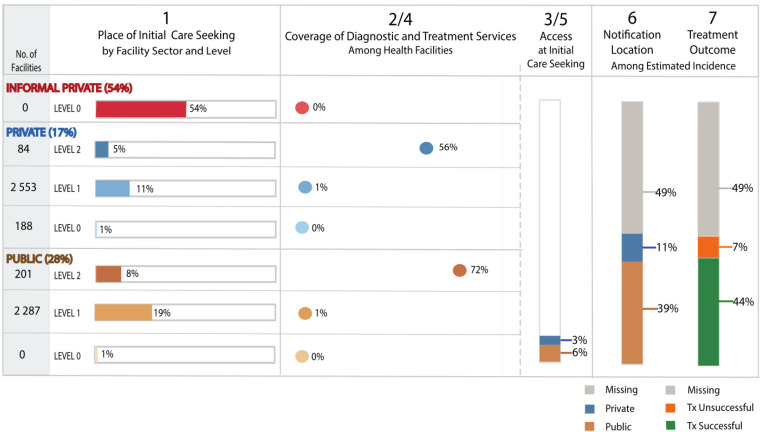
Visual of the patient-pathway analysis for TB services in Cameroon.

A summary of reported care-seeking and availability of TB services by health sector and level is shown in Figure 1. Nationally, 54% of people reported seeking initial care in the informal private sector, 17% in the formal private sector, and 28% in the public sector (Column 1). Coverage of TB testing and treatment services are shown in the next column (Column 2/4), with the proportion of health facilities at each health sector level. In the private sector, an estimated 56% of the 84 Level 2 and above, 1% of the 2553 Level 1, and none of the 188 Level 0 private facilities provided TB services; in the public sector, an estimated 72% of the 201 Level 2 and above and 1% of 2287 Level 1 facilities provided TB services. Overall, an estimated 9% of people had access to TB diagnostic and treatment services at their initial point of care-seeking (Column 3/5).

Access to TB diagnostic and treatment services is concentrated in the public sector in Cameroon, where 6% of people seeking care had access to TB services, with fewer people accessing TB services in the private sector, including faith-based non-profit, private non-profit, and for-profit facilities (3%) (Column 3/5).

In 2018, 23,752 people were notified as starting TB treatment, while the WHO estimated that 47,000 people were ill from TB, corresponding to an estimated 49% of people missing from notification and treatment outcome as shown in columns 6 and 7. Among the estimated people with TB, 11% were notified as starting treatment in the private sector while 39% started in the public sector. Overall, 44% and 7% were notified with a successful and unsuccessful TB treatment outcome, respectively (death or loss to follow up on treatment).

**Figure 2:**
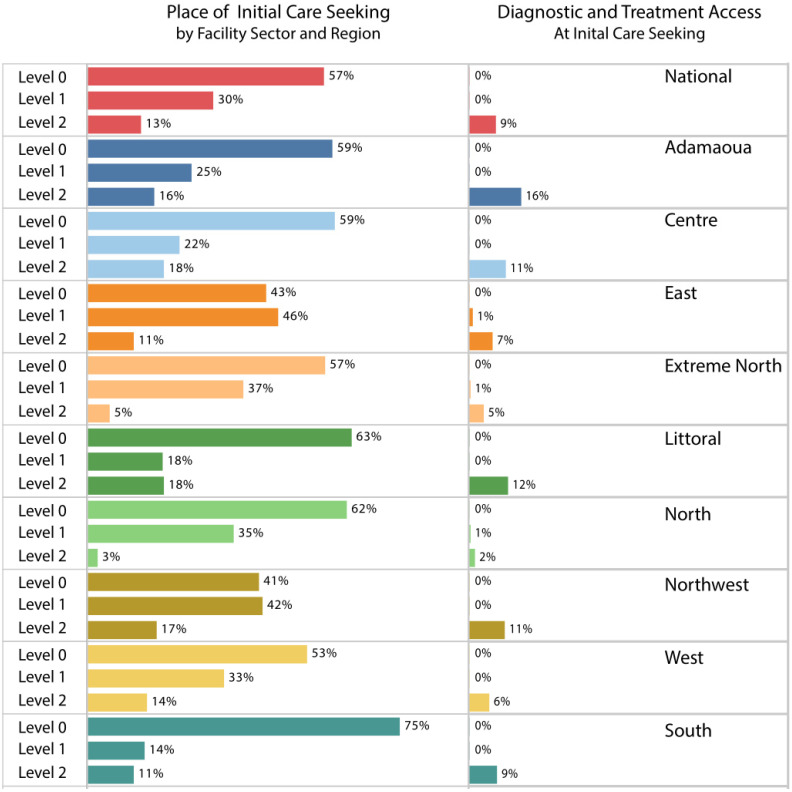
Estimated percentage of people by reported place of initial care-seeking and with access to TB testing and treatment services at initial care-seeking, by health facility level and region.

To further explore the availability of services, we evaluated access by region, where initial care-seeking and access to TB testing and treatment are shown by the level of service (Level 0, 1, or 2) in Figure 2 and by type of service provider (public, private, or informal private, Figure S1). Across all regions, 57% of people sought care initially at Level 0 primarily with informal care providers, such as at pharmacies, stores, and traditional providers; this varied from 41% to 75% across the 10 regions. For people who sought care at health facilities, people seeking care at primary care facilities (Level 1) ranged from 14–46%, while those seeking care at hospitals (Level 2) ranged from 3–18% across the regions. Across all regions, access to TB services at initial care-seeking was low, ranging from 3% to 16%.

**Figure 3:**
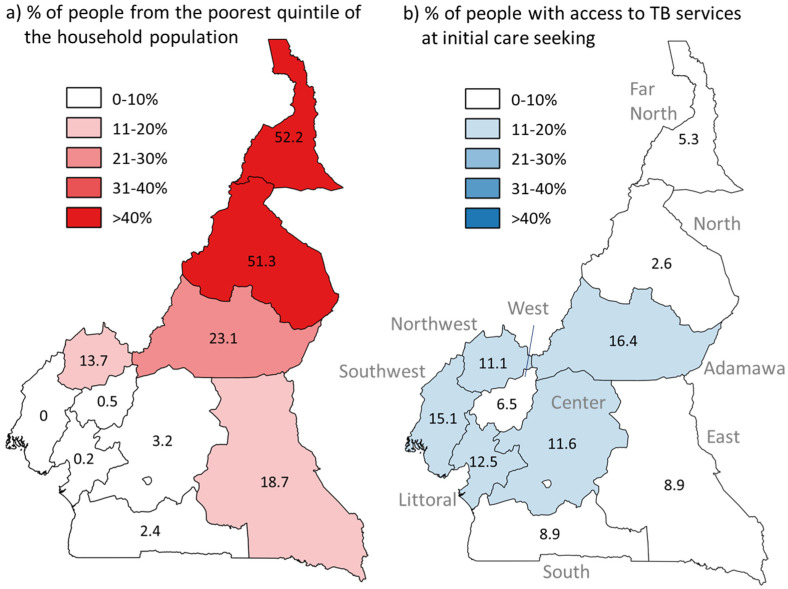
Comparison of (**a**) estimated distribution of people in the lowest quintile of the household population (DHS 2018) and (**b**) estimated percentage of people with access to TB services at initial care-seeking, by geographical region.

In Figure 3, the estimated percentage of people having access to TB services at initial care-seeking is shown by geographic region and compared to the wealth distribution across regions. More than 50% of people living in the Far North and North regions live in households that are in the lowest wealth quintile nationally; these two regions also have the lowest percentage of people who had access to TB services at initial care-seeking based on the patient-pathway analysis, at 5.3% and 2.6% respectively, compared to the national average of 8.9%.

**Figure 4:**
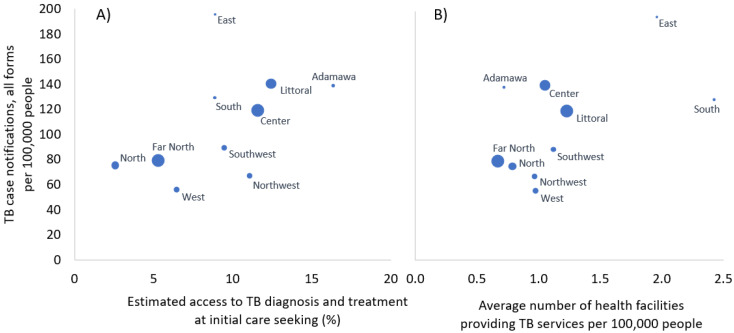
Scatter plots comparing TB case notification rates to the (**A**) average number of health facilities providing TB services and (**B**) estimated access to TB diagnostic and treatment services at initial care-seeking per 100,000 inhabitants; the size of the circles for each region is proportional to the regional populations.

At the regional level, TB case notification rates appear broadly positively correlated with both the number of health facilities providing TB services and access to TB services at initial care-seeking (Figure 4).

## 4. Discussion

Among Cameroonians who sought health services, only a small percentage of the population encountered TB services at their initial point of care, with variability by geographical region (3–16%). Our findings show that TB care-seeking often begins in the informal private sector or at primary care facilities where there are few TB services and limited referral networks. Despite regional variation, the patient-initiated trajectories in all regions had poor overlap with the spatial and institutional distribution of TB care. The lack of availability of TB diagnostic services at the location of initial care-seeking was most acute in the North and Far North regions, where >50% of the population is in the bottom wealth quintile of the country.

The approach to analyze the TB patient pathway that we used here was initially described by Hanson et al. in 2017 [13]. At that time, the PPA was assessed for five countries: Ethiopia, Kenya, Indonesia, Pakistan, and the Philippines. Across these five countries, the combined patient-pathway analysis indicated that 24% of people had access to diagnosis at initial care-seeking and 25% had access to TB treatment. There were widely varying levels of initial care-seeking in the private sector across countries, ranging from 24% (Ethiopia) to 85% (Pakistan), as well as highly disparate care-seeking at the sub-national level within countries, highlighting the need for locally tailored approaches to TB service delivery. Since the initial publication of the PPA results from these five countries in 2017, we could only find a single publication from Taiwan using a patient-pathway analysis of individual patient healthcare data to assess the alignment of service provision with care-seeking [21]. Our results help demonstrate the feasibility of conducting a PPA in a high TB burden country to help inform the national TB response.

The World Health Organization recommends that all people evaluated for TB receive a rapid molecular diagnostic test [22]. Of the approximately 5300 facilities in the 2017 Health Facilities List, 256 (5%) of these offered TB diagnostic testing, and there were 24 sites that provided Xpert MTB/RIF (Cepheid, Sunnyvale, CA, USA) testing, with the remaining 232 TB testing sites offering smear microscopy rather than a rapid molecular test; by 2020, there were 28 Xpert testing sites and 22 sites providing TB LAMP (Eiken Chemical, Tokyo, Japan) testing. The limited laboratory capacity for rapid, sensitive TB diagnosis suggests the need to expand access to better diagnostic testing by increasing these facilities, as well as by scaling up specimen transport between facilities to provide more widespread access to molecular testing. The 2018 Cameroon Health Facility Assessment reported that the availability of TB services is significantly less than for HIV and malaria, for example, as an estimated 93% of health facilities offer HIV services, and 99% offer malaria services [23]. In contrast to HIV, malaria, and COVID-19, there is not yet any currently available rapid, inexpensive point-of-care diagnostic test recommended for TB, although new point-of-care diagnostics show promise, including urine LAM testing [24].

This analysis had several limitations. The PPA approach has inherent limitations based on the quality of the data sources used [12]. In this study, for initial care-seeking, we used a behavior proxy (e.g., parents who had sought care for a child with fever) because granular TB-specific care-seeking information for both adults and children was not available for Cameroon. This proxy is recommended by Hanson et al. when TB-specific care-seeking information is not available, since general care-seeking behavior and TB care-seeking behavior have been found to be similar in some countries where both data sources are available [13]. Another limitation is that the health facility master list used in this analysis included only 90% of the facilities in the mapping of facilities by the Ministry of Health in 2016, and the number of facilities has likely shifted slightly since that time. However, this is unlikely to impact the proportions reported, since the distribution of facilities by region and type of facility is similar in both sources, and all of the TB diagnostic and treatment centers where people could access TB services at initial care-seeking appear on the health facility list. An additional limitation is that for one of the ten regions (Southwest), we used data from DHS 2011 rather than 2018 since the DHS 2018 data collection in the region was limited due to armed conflict in the area. We also performed the PPA using the DHS 2011 data and obtained similar results at the national level (13% vs. 9% national access to TB services at initial care-seeking in 2011 vs. 2018), as well as for the other nine regions, indicating that the use of the 2011 data for one region is unlikely to cause a significant change in the overall findings reported here, especially as policies are set at the national rather than regional level. This analysis explores the alignment between initial care-seeking and availability of TB services for people sick with TB, and implicit in this analysis (and in all of the gap models) is the idea that TB disease is symptomatic. This premise is increasingly contested, since many people with TB disease do not report symptoms [2]. These models also do not typically include services for TB prevention, including the provision of TB preventive therapy and implementation of effective infection control measures, which are also essential strategies for ending TB [11]. Finally, the actual gap between people sick with TB and those who access TB treatment is difficult to know with accuracy, due in large part to the challenge to estimate TB incidence, as has been described in detail elsewhere [17,25,26].

Further research is ongoing to assess the median cost and duration of health-seeking to determine how the barriers to access described here affect specific groups at risk. As the risk of TB is not homogeneously distributed in the population, future analyses should focus on those most at risk for TB. Figure 3 highlights the mismatch between distribution of TB services and place of residence of those in the lowest socioeconomic quintile, who have added vulnerability to TB for a variety of reasons, including malnutrition [27]. The implications of lack of access in terms of financial costs and tendency to delay and to forgo diagnostic testing for TB symptoms merit research and mitigation efforts.

## 5. Conclusions

While people with TB who are effectively linked to care and notified to the National TB Program in Cameroon have generally good treatment outcomes, most people (91%) do not report attending health facilities with TB services in their initial encounters with the health system. A lack of access at the first point of contact likely leads to delayed diagnosis, additional patient costs, and drop-outs along the patient pathway, contributing to the low estimated TB treatment coverage for the country (53% in 2019). This analysis suggests that access to services for people with TB may be improved by providing services more equitably across the health system. Currently, there are various activities being conducted in Cameroon to address these gaps, including research to better understand the barriers to care, scale-up of the number of sites performing TB testing, and strengthening of the specimen transport system to enable people attending primary care sites to be tested for TB.

## Figures and Tables

**Table 1:** Data sources for Cameroon patient-pathway analysis (PPA) of tuberculosis (TB) services.

PPA Component	Data Source
Number of health facilities	2017 Health Facility List
Place of initial care-seeking	2018 Demographic and Health Survey
Place of care-seeking for child with fever (proxy for TB care-seeking)	
Tuberculosis diagnostic and treatment services coverage	2018 National TB Program data
Tuberculosis treatment location	
Tuberculosis case notification and treatment success	

## Data Availability

The data used in this study are available on reasonable request from the Cameroon National TB Program. The data are not publicly available due to privacy reasons.

## Supplementary Materials

The following are available online at https://www.mdpi.com/article/10.3390/tropicalmed6040171/s1, Table S1: Health facility coding for PPA in Cameroon; Figure S1: Proportion of people by reported place of initial care seeking and with access to TB testing and treatment services at initial care seeking, by health facility type and region.
