# Supplementary material for: Patient-Pathway Analysis of Tuberculosis Services in Cameroon
Source: Trop Med Infect Dis. 2021 Sep 22;6(4):171. doi: 10.3390/tropicalmed6040171 (PMC8544654; doi:10.3390/tropicalmed6040171)
Supplement: Supplementary file 1 [file tropicalmed-06-00171-s001.zip › resupplementary-1336640.pdf]

Supplementary file.

Patient-pathway analysis of tuberculosis services in Cameroon.

Table S1. Health facility coding for Patient-pathway analysis (PPA) in Cameroon

	**Categorization**	
**Data Source, Health Facility Type**	**Health Facility Sector**	**Health Facility Level**
**2018 Demographic and Health Survey**		
Government hospital	Public	2
Government health center	Public	1
Health worker/field worker	Public	0
Other public	Public	0
Private confessional hospital, clinic	Private	2
Lay private hospital/clinic	Private	2
Confessional health center	Private	1
Medical cabinet	Private	0
Other private medical	Private	0
Pharmacy	Informal Private	0
Informal seller of drug	Informal Private	0
Traditional practitioner	Informal Private	0
NGO	Informal Private	0
Store	Informal Private	0
Community relay/worker	Informal Private	0
Other	Informal Private	0
**Cameroon Health Facility List and National TB Program data**		
General Hospital*	Public	3
Central Hospital*	Public	3
Regional Hospital*	Public	3
District Hospital	Public	2
Integrated health center (CSI)	Public	1
Sub-divisional medical center (CMA)	Public	1
Prison†	Public	1
Hospital	Private	2
Clinic	Private	1
Health center	Private	1
Medical cabinet	Private	0

*The Level 3 public structures in the health facility list have been merged with the Level 2 public structures for the analysis to facilitate alignment with the DHS care seeking data. †The NTP-supervised facilities are a sub-set of the Health Facility list, with the exception of prisons; the NTP supervises 4 prisons that have both TB diagnostic and treatment services and are not included on the Health Facility List.

Region	Type of facility	Care seeking %	Dx/Tx access %
National	Informal Private	32%	0%
	Private	40%	3%
	Public	29%	6%
Adamawa	Informal Private	23%	0%
	Private	38%	2%
	Public	39%	14%
Centre	Informal Private	21%	0%
	Private	55%	3%
	Public	24%	9%
East	Informal Private	19%	0%
	Private	48%	2%
	Public	33%	7%
Far North	Informal Private	54%	0%
	Private	10%	2%
	Public	36%	3%
Littoral	Informal Private	15%	0%
	Private	64%	3%
	Public	21%	9%
North	Informal Private	60%	0%
	Private	9%	0%
	Public	31%	2%
Northwest	Informal Private	29%	0%
	Private	26%	4%
	Public	46%	7%
West	Informal Private	21%	0%
	Private	59%	3%
	Public	20%	3%
South	Informal Private	39%	0%
	Private	36%	2%
	Public	25%	7%

Figure S1. Proportion of people by reported place of initial care seeking and with access to TB testing (Dx) and treatment (Tx) services at initial care seeking, by health facility type and region
